# Risk factors for unstructured treatment interruptions and association with survival in low to middle income countries

**DOI:** 10.1186/s12981-016-0109-8

**Published:** 2016-07-11

**Authors:** James H. McMahon, Tim Spelman, Nathan Ford, Jane Greig, Anita Mesic, Charles Ssonko, Esther C. Casas, Daniel P. O’Brien

**Affiliations:** Infectious Diseases, Monash University and Alfred Hospital, Melbourne, Australia; Burnet Institute, Centre for Population Health, Melbourne, Australia; Infectious Disease Epidemiology and Research, University of Cape Town, Cape Town, South Africa; Division of Infectious Diseases, Imperial College London, London, UK; Manson Unit, Médecins Sans Frontières, London, UK; Public Health Department, Médecins Sans Frontières, Amsterdam, The Netherlands; Department of Medicine and Infectious Diseases, Royal Melbourne Hospital, University of Melbourne, Melbourne, Australia

**Keywords:** Unstructured treatment interruption, Antiretroviral therapy, Resource limited settings, Clinical outcomes, Survival, Epidemiology

## Abstract

**Background:**

Antiretroviral therapy (ART) treatment interruptions lead to poor clinical outcomes with unplanned or unstructured TIs (uTIs) likely to be underreported. This study describes; uTIs, their risk factors and association with survival.

**Methods:**

Analysis of ART programmatic data from 11 countries across Asia and Africa between 2003 and 2013 where an uTI was defined as a ≥90-day patient initiated break from ART calculated from the last day the previous ART prescription would have run out until the date of the next ART prescription. Factors predicting uTI were assessed with a conditional risk-set multiple failure time-to-event model to account for repeated events per subject. Association between uTI and mortality was assessed using Cox proportional hazards, with a competing risks extension to test for the influence of lost to follow-up (LTFU).

**Results:**

40,632 patients were included from 11 countries across 33 sites (17 Africa, 16 Asia). Median duration of follow-up was 1.61 years (IQR 0.54–3.31 years), 3386 (8.3 %) patients died, and 3453 (8.5 %) were LTFU. There were 14,817 uTIs, with 10,162 (25 %) patients having more than one uTI. In the adjusted model males were at lower risk of uTI (aHR 0.94, p < 0.01, and age 20–59 was protective compared to <20 years (20–39 years aHR 0.87, p < 0.01; 40–59 years aHR 0.86, p < 0.01). Preserved immune function, as measured by higher CD4 cell count, was associated with a reduced rate of uTI compared to CD4 <200 cells/μL (CD4 200–350 cells/μL aHR 0.89, p < 0.01; CD4 >350 cells/μL aHR 0.87, p < 0.01), whereas advanced clinical disease was associated with increased uTI rate (WHO stage 3 aHR 1.10, p < 0.01; WHO stage 4 aHR 1.21, p < 0.01). There was no relationship between uTI and mortality after adjusting for disease status and considering LTFU as a competing risk.

**Conclusions:**

uTIs were frequent in people in ART programs in low-middle income countries and associated with younger age, female gender and advanced HIV. uTI did not predict survival when loss to follow-up was considered a competing risk. Further evaluation of uTI predictors and interventions to reduce their occurrence is warranted.

## Background

Unstructured treatment interruptions (uTIs) are patient-initiated interruptions to antiretroviral therapy (ART) and a reality of routine clinical care. They often occur as a result of treatment fatigue or in an attempt to minimize side effects, and can be precipitated by different social and behavioural factors [1]. Distinct from medically guided interruptions performed for medical reasons such as toxicity, drug interactions or pregnancy, uTIs are associated with increased risk of mortality, opportunistic infections, virological failure and drug resistance in high-income countries [2–5].

Several systematic reviews have documented high rates of patient attrition along the cascade of care from HIV testing to long-term virological suppression [6–8]. More recently, it has been acknowledged that patient engagement in care is not a linear pathway; rather, patients may disengage and reengage with care along the care pathway, a phenomenon recently described as churning in and out of care [9]. uTIs are a common yet inconsistently reported phenomenon that can be seen as contributing to such churning [1]. This study describes the frequency and risk factors for uTIs and the association of uTI with survival in Médecins Sans Frontières (MSF) ART programmes in 33 sites across Asia and Africa.

## Methods

### Study setting and population

This is a retrospective observational multi-site cohort study of ART treatment sites at hospitals and peripheral health clinics in MSF supported programs from 11 countries in Africa and Asia. All sites diagnosed HIV associated clinical conditions using World Health Organization (WHO) criteria [10] and provided free treatment and laboratory monitoring via protocols standardized across MSF programmes [11]. Eligibility criteria for ART were standardized and based on contemporaneous WHO guidelines, with first-line regimens consisting of a backbone of two nucleoside reverse-transcriptase inhibitor (NRTIs) plus a non-nucleoside reverse-transcriptase inhibitor (NNRTI) [10]. Due to resource limitations, initiation of ART in one country between 2007 and 2013 was based on lower CD4 criteria ranging from 100–250 cells/μL. Adherence counselling was provided prior to initiation and while on ART, with laboratory monitoring typically including CD4 cell counts (HIV viral load was not routinely available in most sites during the period of observation).

Routine clinical data was collected on standardised forms at each clinic visit by treating clinicians and entered into a standardized electronic database [Follow-up and Care of HIV Infection and AIDS (FUCHIA), Epicentre, Paris]. Data were extracted from individual program databases, de-identified and collated at MSF-Operational Centre Amsterdam.

This study examined all adult patients (≥15 years) with at least 3 months of follow-up data who commenced ART between 28 March 2003 and 11 January 2013 and included the following variables: gender, age, marital status, region (Asia or Africa), date of initiation (2003–2005 or after 2005), CD4 cell count and WHO stage at ART initiation. uTI was defined as a ≥90 day patient initiated break from ART where people had a recorded resumption of treatment after an ART supply would have run out. uTI was calculated from the last day the previous ART prescription ran out until the date of the next ART prescription. Individuals were considered lost to follow-up (LTFU) if there was no record of further attendance >90 days after the ART supply would have run out. Individuals could be recorded as died by contact from family members or peer networks reporting back to the site or if they had died while in direct care of the programme. Notably there is no consensus definition for an uTI with a systematic review reporting durations from 24 h to 1 year (median duration 150 days). A common working definition for LTFU is >90 days since last ART pick up and is recommended in current WHO consolidated guidelines [12]. We elected to apply a similar duration for uTI but instead start the time period from the last known supply of ART would have run out.

### Statistical analyses

Categorical variables were summarised using frequency and percentage. The number of individuals with 1, 2 or >2 uTIs were reported and stratified by survival or LTFU status. A conditional risk-set time-to-event model appropriate for ordered, multiple failure time data such as treatment interruptions was used to estimate hazard ratios for baseline variables predicting uTI and therefore was a model that accounted for multiple uTIs per subject. Tests for interaction were performed in the adjusted models. The association between uTI and mortality was assessed in a separate Cox proportional hazards regression that was adjusted for markers of disease severity, namely WHO stage and CD4 cell count at ART initiation. Hazard proportionality was assessed through analysis of scaled Schoenfeld residuals. A competing risks regression was used to assess the magnitude, if any, of LTFU as a competing risk for observing an association between uTI and survival. For all analyses, p < 0.05 was considered significant. All analyses were performed using Stata version 13 (StataCorp, College Station, Texas, USA).

### Ethics

This study met the standards set by the independent MSF Ethics review board for retrospective analyses of routinely collected programmatic data [13] and was approved by the Alfred Hospital Human Research and Ethics Committee (Number 183/14).

## Results

This study included 40,632 people from nine countries in Africa and two countries in Asia across 33 sites (17 in Africa and 16 in Asia). For the 10,162 people who experienced an uTI: 4835 (47.6 %) were female, 481 (4.7 %) were under 20 years of age, 7908 (77.8 %) were classified as WHO clinical stage 3 or 4, 3864 (38.0 %) had CD4 cell counts <200 cells/μL, 4613 (45.6 %) were not married and 8166 (80.4 %) were from Asian sites. (Table 1). Median duration of follow-up since commencement of ART was 1.61 years (IQR 0.54–3.31 years) in the entire cohort, 3386 (8.3 %) patients died and 3452 (8.5 %) were LTFU. The 10,162 (25.0 %) individuals experienced 14,817 uTIs of ≥90 days with 7030 (17.3 %) people having one uTI, 2112 (5.2 %) having two uTIs and 1020 (2.5 %) having more than two uTIs (Table 2). In the subset of patients experiencing an uTI the median time to the first uTI was 1.9 years.

**Table 1:** Cohort characteristics at baseline

Predictor	Level	No treatment interruption n (%)	≥90 day treatment interruption n (%)
		n = 30,472	n = 10,162
Sex	Female	14,785 (48.5)	4835 (47.6)
	Male	15,675 (51.4)	5326 (52.4)
Age (years)	<20	1601 (5.3)	481 (4.7)
	20–39	18,408 (60.4)	6217 (61.2)
	40–59	7181 (23.6)	1877 (18.5)
	60+	318 (1.0)	67 (0.7)
WHO stage at ART initiation	1	2704 (8.9)	980 (9.6)
	2	2862 (9.4)	1063 (10.5)
	3	15,317 (50.3)	5083 (50.0)
	4	9001 (29.5)	2825 (27.8)
CD4 count at ART initiation (cells/μL)	<200	13,182 (45.3)	3864 (38.0)
	200–350	4000 (13.1)	1348 (13.3)
	>350	1304 (4.3)	450 (4.4)
Marital status	Not married	13,517 (44.4)	4613 (45.6)
	Married	15,945 (52.3)	5343 (52.6)
Region	Africa	6189 (20.3)	1996 (19.6)
	Asia	24,281 (79.7)	8166 (80.4)

Missing data <5 % for all apart from; age (9.7 % no uTI, 15.0 % uTI), and CD4 count (37.3 % no uTI, 44.3 % uTI)

*uTI* unstructured treatment interruption

**Table 2:** Number of people with and without unstructured treatment interruptions stratified by death and lost to follow-up status (14,817 uTIs in 40,632 people)

Unstructured treatment interruptions (n)	People n (%)	Died n (%)	Lost to follow-up n (%)
0	30,470 (75.0)	3046 (10.0)	2579 (8.5)
1	7030 (17.3)	257 (3.7)	587 (8.3)
2	2112 (5.2)	34 (1.6)	191 (9.0)
>2	1020 (2.5)	9 (0.9)	95 (9.3)
Total	40,632	3386	3452

Unadjusted and adjusted risk factors at baseline for uTI are presented in Table 3. In the adjusted model male gender was associated with a lower rate of an uTI compared with female (aHR 0.94, p < 0.01), and individuals from 20 to 59 years of age were at lower risk of uTI (20–39 years aHR 0.87, p < 0.01; 40–59 years aHR 0.86, p < 0.01), as compared to those <20 years. More advanced clinical disease (WHO stage 3 aHR 1.10, p < 0.01; stage 4 aHR of 1.21, p < 0.01) was predictive of increased rate of uTI. Preserved immune function, CD4 cell count 200–350 or >350 cells/μL, was protective of uTI as compared to CD4 <200 cells/μL in the same adjusted model (CD4 200–350 cells/μL aHR 0.89, p < 0.01; CD4 >350 cells/μL aHR 0.87, p < 0.01). Marital status (HR 0.97, p = 0.18) and later date of initiation (HR 1.07, p = 0.12) was not protective of uTI, while people from Asian countries were less likely to experience an uTI when compared with those from African nations (aHR 0.82, p < 0.01).

**Table 3:** Unadjusted and adjusted models for predictors of ≥90 day unstructured treatment interruption across 33 MSF sites providing ART care

Predictor	Level	Unadjusted hazard ratio (95 % CI)	p value	Adjusted hazard ratio (95 % CI)	p value
Sex	Female	Ref.		Ref.	
	Male	0.94 (0.90–0.98)	<0.01	0.94 (0.90–0.98)	<0.01
Age	<20	Ref.		Ref.	
	20–39	0.81 (0.74–0.89)	<0.01	0.87 (0.79–0.96)	<0.01
	40–59	0.79 (0.71–0.87)	<0.01	0.86 (0.78–0.96)	<0.01
	60+	0.86 (0.67–1.11)	0.26	1.07 (0.82–1.39)	0.61
WHO stage at ART initiation	1	Ref.		Ref.	
	2	1.07 (0.98–1.17)	0.15	1.03 (0.95–1.13)	0.47
	3	1.12 (1.05–1.21)	<0.01	1.10 (1.02–1.18)	<0.01
	4	1.23 (1.14–1.33)	<0.01	1.21 (1.12–1.30)	<0.01
CD4 count at ART initiation* (cells/μL)	<200	Ref.		Ref.	
	200–350	0.87 (0.82–0.93)	<0.01	0.89 (0.83–0.95)	<0.01
	>350	0.86 (0.78–0.95)	<0.01	0.87 (0.78–0.96)	<0.01
Marital status	Not married	Ref.		Ref.	
	Married	0.97 (0.93–1.01)	0.18	1.02 (0.97–1.06)	0.48
Region	Africa	Ref.		Ref.	
	Asia	0.79 (0.75–0.83)	<0.01	0.82 (0.78–0.87)	<0.01

* Individuals with missing CD4 counts were modelled as having missing data and not excluded from the analysis. Compared to the reference group (CD4 count <200) the aHR for those with missing CD4 data was 0.99 (0.94–1.03) p = 0.57

Associations between uTI and mortality were assessed in an adjusted model controlling for markers of disease severity namely WHO clinical stage and CD4 cell counts at baseline (Table 4). In this model treatment interruption was protective against mortality (aHR 0.21, p < 0.01), with advanced clinical disease (WHO stage 3 aHR 2.41, p < 0.01; stage 4 aHR of 4.31, p < 0.01) predicting mortality, and preserved immune function having a lower rate of mortality (CD4 200–350 cells/μL aHR 0.55, p < 0.01; CD4 >350 cells/μL aHR 0.41, p < 0.01 as compared to CD4 <200 cells/μL). When this model then incorporated LTFU as a competing risk the protective effect of treatment interruption for survival disappeared (aHR 0.95, p = 0.18). Advanced WHO stage at baseline (WHO stage 3 aHR 2.50, p < 0.01; stage 4 aHR of 4.50, p < 0.01) and lower CD4 cell count still predicted decreased survival (CD4 200–350 cells/μL aHR 0.54, p < 0.01; CD4 >350 cells/μL aHR 0.40, p < 0.01 as compared to CD4 <200 cells/μL).

**Table 4:** Unadjusted and adjusted models for predictors of mortality across 33 MSF sites providing ART care

Model outcome	Level	Mortality		Mortality with lost to follow-up as a competing risk	
Predictor		Adjusted hazard ratio (95 % CI)	p value	Adjusted hazard ratio (95 % CI)	p value
Treatment interruption		0.21 (0.18, 0.23)	<0.01	0.95 (0.89, 1.02)	0.18
WHO stage at ART initiation	1	Ref.		Ref.	
	2	1.06 (0.81, 1.40)	0.67	1.06 (0.80, 1.40)	0.68
	3	2.41 (1.95, 2.98)	<0.01	2.50 (2.02, 3.09)	<0.01
	4	4.31 (3.48, 5.33)	<0.01	4.50 (3.64, 5.56)	<0.01
CD4 count at ART initiation (cells/μL)	<200	Ref.		Ref.	
	200–350	0.55 (0.48, 0.64)	<0.01	0.54 (0.47, 0.62)	<0.01
	>350	0.41 (0.30, 0.55)	<0.01	0.40 (0.29, 0.53)	<0.01

## Discussion

Unstructured TIs were frequent in this large cohort combining data from multiple ART sites across countries in Africa and Asia. One quarter of individuals experienced uTI and one quarter of the interrupting group experienced more than two treatment interruptions. These findings have the potential for considerable additional HIV associated morbidity and additional new HIV infections. This analysis identified younger age, female gender and more advanced HIV (higher WHO clinical stage and lower CD4 cell count) at baseline being predictive of uTI.

The finding of younger age predicting uTI has been reported previously from high-income countries [14, 15] with limited data available from low-middle income countries (LMICs). A recently reported Nigerian cohort found no association between age and uTI in a multivariate analysis [16] and an additional report described younger age predicting uTI in French Guyana [17]. Furthermore, younger patients have been found to have increased rates of loss to follow-up from ART programmes in LMICs, and thus it would seem plausible that they may also be more likely to self-interrupt ART [18, 19].

Previous reports describing CD4 cell count as a risk factor for uTI have varied with some studies reporting higher CD4 cell count and others lower CD4 cell count predicting uTI again mostly from high-income countries [2, 14, 20]. In LMICs, baseline CD4 above 350 cells/μL were found to be a potent predictor of uTI in a program providing free ART in Nigeria [16] and a report from South Africa described CD4 cell counts above 200 cells/μL predicting default from ART program and of these defaulters, those with higher CD4 cell count were less likely to resume ART [21]. Another report from South Africa described no association with baseline CD4 cell count [22] and a Ugandan study associated baseline CD4 cell count above 350 cells/μL with 12 month interruptions [23]. While differing from these LMIC studies, the finding of lower CD4 cell count and advanced clinical stage being associated with uTI from this analysis may mirror the mixed findings of high-income country studies. The association of uTI with advanced HIV may relate to patient health seeking behavior, health literacy and adherence characteristics. An association between lower CD4 cell count and lower adherence has been reported in some studies [24, 25] and patients self-report of ‘feeling sick’ has been described as a common barrier to adherence [26], however this relationship with CD4 cell count is not consistently reported [27].

The finding that women have increased likelihood of uTI in this study has also been reported in some high-income countries studies but not others [2, 15, 20, 28], while in LMICs one study reported no association between uTI and gender [16] and another that females are at decreased risk of uTI [22]. While these findings are mixed, another consideration is the large amount of data describing male gender as an independent risk factor for becoming LTFU or dying in LMIC ART programmes [29–33]. The finding that women have increased likelihood of uTI in this large analysis may reflect that women are more likely than men to return to the clinic at a later date and recommence treatment whereas men may remain LTFU. It may also reflect women intermittently in care due to competing priorities such as caring for children, especially if they have entered ART care via antenatal or prevention of mother to children transmission services.

The reasons for the association of fewer uTIs and receiving care in Asian programme sites are not clear, but may involve population differences in social, economic, genetic and contextual factors. These include the ease of access to care, rural program location, unstable contexts, resource utilization within projects, and relative prevalence of adverse effects to ART medications. In addition, site specific factors that have not been assessed may be important predictors of uTI at a local level. Further research to determine local factors may also lead to the design of tailored interventions to improve the proportion of people receiving continuous treatment. Interventions using short messaging service (SMS) reminders to improve ART adherence have been shown to decrease the number of treatment interruptions over 48 h [34]. This is consistent with another large randomized trial in Kenya that demonstrated how weekly SMS reminders can improve adherence to ART [35]. Additionally, a study of 63 subjects in Uganda found benefit in weekly SMS reminders to decrease 2 and 4 day uTIs although did not see the same effect of SMS reminders on uTIs triggered by late or missed doses [36]. Other studies identifying predictors of uTI have suggested interventions such as people recently initiating ART or with a history of injection drug use be targeted [15, 21]. However, randomised trials of interventions for people experiencing uTIs of 3 months or more are not reported.

When examining uTI as a predictor of mortality it was found to be a potent predictor of survival with an approximate 80 % reduction in the risk of death while controlling for clinical stage and CD4 cell count. Initially this could be seen as a surprising finding when considering the adverse effects of structured interruption studied at a time when ART was considered potentially harmful at higher CD4 cell counts and ultimately found to pose increased risk for death and morbidity [37]. Importantly, when LTFU was considered as a competing risk in the survival analysis the protective effect of treatment interruption on survival disappeared. This suggests that individuals who interrupt treatment are not more likely to survive as the initial model presented, but many of these people interrupting therapy and considered survivors for the purpose of the analysis were in fact LTFU. Meta-analyses reporting the proportion of people LTFU in LMICs that had died estimated that 39 % of people reported as LTFU ultimately die [38], and also observed the proportion of the LTFU population that died decreased as the proportion LTFU increased [39]. Furthermore individuals included in our analysis who experienced uTI were more likely to have advanced disease with lower CD4 cell count and increased WHO clinical stage at baseline. These factors raise concerns that people having uTIs and subsequently LTFU are at risk of decreased survival once they disengage from ART care. Observational studies from high-income countries include the Swiss cohort study that reported short interruptions of 3 months or less not predicting survival [40] but then later reported an increased risk of death with interruptions over 1 month [5]. An additional European cohort also reported an association between treatment interruptions over 3 months and an increased risk of the combined outcome of AIDS and death [2]. Results from our analysis have important implications for other cohorts which should consider LTFU as a competing risk when attempting to link uTI with mortality.

This study has several limitations including the potential for unmeasured confounding to impact on the associations between baseline factors with uTI and also uTI with survival. Another limitation is that over a third of baseline CD4 counts were missing, which potentially interferes with the documented CD4 count associations. This missing data is most likely related to earlier practices at some sites where CD4 testing was not available or individuals that met clinical criteria for ART initiation were not tested for CD4. This is supported by the fact that individuals with missing baseline CD4 data had the same rate of uTI as those with CD4 counts below 200. In addition this study reports over 8 % of individuals as LTFU of which a proportion will have died. A more accurate ascertainment of death across the whole population including those LTFU may have impacted our findings. It should also be noted that minimal HIV viral load data was available as virological monitoring was not standard of care for the duration of this study and hence this was not included in the analysis.

## Conclusions

Unstructured treatment interruptions are common in ART programmes across a range of LMIC settings. Interpreting the impact of uTI on survival needs to take into account the number of individuals LTFU and further studies examining the relationship between uTI and survival are needed. Defining predictors of uTI and interventions to minimise uTI will be necessary for ART programs to maximise treatment outcomes.
